# Duct of Luschka Bile Leak With Formation of Biloma Post-Cholecystectomy: A Case Study

**DOI:** 10.7759/cureus.72639

**Published:** 2024-10-29

**Authors:** Jamie McDermott, Nima Sadeghi, Dayna Telken, Imtiaz Ahmed

**Affiliations:** 1 Medical School, Midwestern University Arizona College of Osteopathic Medicine, Glendale, USA; 2 Radiology, Midwestern University Arizona College of Osteopathic Medicine, Glendale, USA; 3 Gastroenterology, Midwestern University Arizona College of Osteopathic Medicine, Glendale, USA; 4 Radiology, Tempe St. Luke's Hospital, Tempe, USA

**Keywords:** bile leak, biloma, cholecystectomy, duct of luschka, luschka, post-cholecystectomy bile leak

## Abstract

A rare complication of laparoscopic cholecystectomy is a biloma, which usually develops as a result of the dissection of non-visible, abnormal ducts of Luschka. This anatomical variation in the bile ducts was initially overlooked within the biliary tree. While it generally holds minimal clinical significance, it may occasionally lead to bile leakage following cholecystectomy. In most cases, this complication is self-limiting and resolves without the need for further intervention. We present a case in which a patient developed a post-cholecystectomy biloma, which formed as a result of a bile leak from the ducts of Luschka. The initial workup included a hepatobiliary iminodiacetic acid (HIDA) scan, also called cholescintigraphy or hepatobiliary scintigraphy, which was used to determine and confirm the source of the leak. Computed tomography (CT) of the abdomen revealed fluid accumulation around the liver and in the cystic duct, as well as fluid collection near the surgical site, which suggests the possibility of an abscess. Management of bile leaks usually involves nonsurgical interventions like endoscopic biliary stent placement. When bilomas are present, treatment typically involves percutaneous drainage, as in this case. However, if conservative management fails, surgical intervention, such as exploratory laparotomy, may become necessary. This report further helps to understand the clinical significance of hepatic bile duct variation and complications of laparoscopic cholecystectomy.

## Introduction

Laparoscopic cholecystectomy is among the most frequently performed surgical procedures. However, significant anatomical variability in the biliary system predisposes patients to both intraoperative and postoperative complications. During the procedure, the cystic duct is ligated to facilitate the removal of the gallbladder. Bile leaks represent a common complication, ranging from clinically insignificant to more serious events. These leaks often originate from the cystic duct stump but may also arise from small ducts within the gallbladder bed, referred to as subvesical bile ducts or the ducts of Luschka [1].

The ducts of Luschka, first described by German anatomist Hubert von Luschka (1820-1875), are small, variable bile ducts measuring approximately 1-2 mm in diameter [1]. They arise from the inferior aspect of the right hepatic lobe, course along the gallbladder fossa, and typically drain into the extrahepatic bile ducts [1,2]. While initially considered rare anomalies occurring in about 4% of the population, more recent studies have demonstrated their presence in up to 70% of cholecystectomy specimens [3-6]. These ducts include variations such as accessory and segmental subvesical bile ducts, which are more common, while aberrant subvesical and hepaticocholecystic bile ducts are rarer [2,4]. Injury to the ducts of Luschka during cholecystectomy is uncommon, with an incidence reported between 0.5% and 2% [1,4,7]. Such injuries typically occur when dissection in the gallbladder fossa is too deep. Bile leaks that arise from these ducts can result in the formation of a biloma, a bile collection, or an abscess within the abdomen. 

Diagnosing bile duct injuries typically requires a combination of imaging modalities and sometimes intervention, depending on the clinical presentation. Endoscopic retrograde cholangiopancreatography (ERCP) and magnetic resonance cholangiopancreatography (MRCP) are the most commonly used diagnostic tools for direct visualization, while CT and ultrasound are useful in identifying complications. Additionally, a hepatobiliary iminodiacetic acid (HIDA) scan uses nuclear medicine to confirm bile leaks. The management of bile duct injuries necessitates a multidisciplinary approach involving biliary endoscopists, interventional radiologists, and hepatobiliary surgeons. Treatment strategies depend on the severity and timing of the injury, the extent of the bile leak, and the presence of a biloma. Surgical interventions may include lavage of the abdominal cavity, closure of the duct of Luschka, and intraoperative cholangiography to confirm the integrity of the biliary tree [7].

## Case presentation

**Figure 1:**
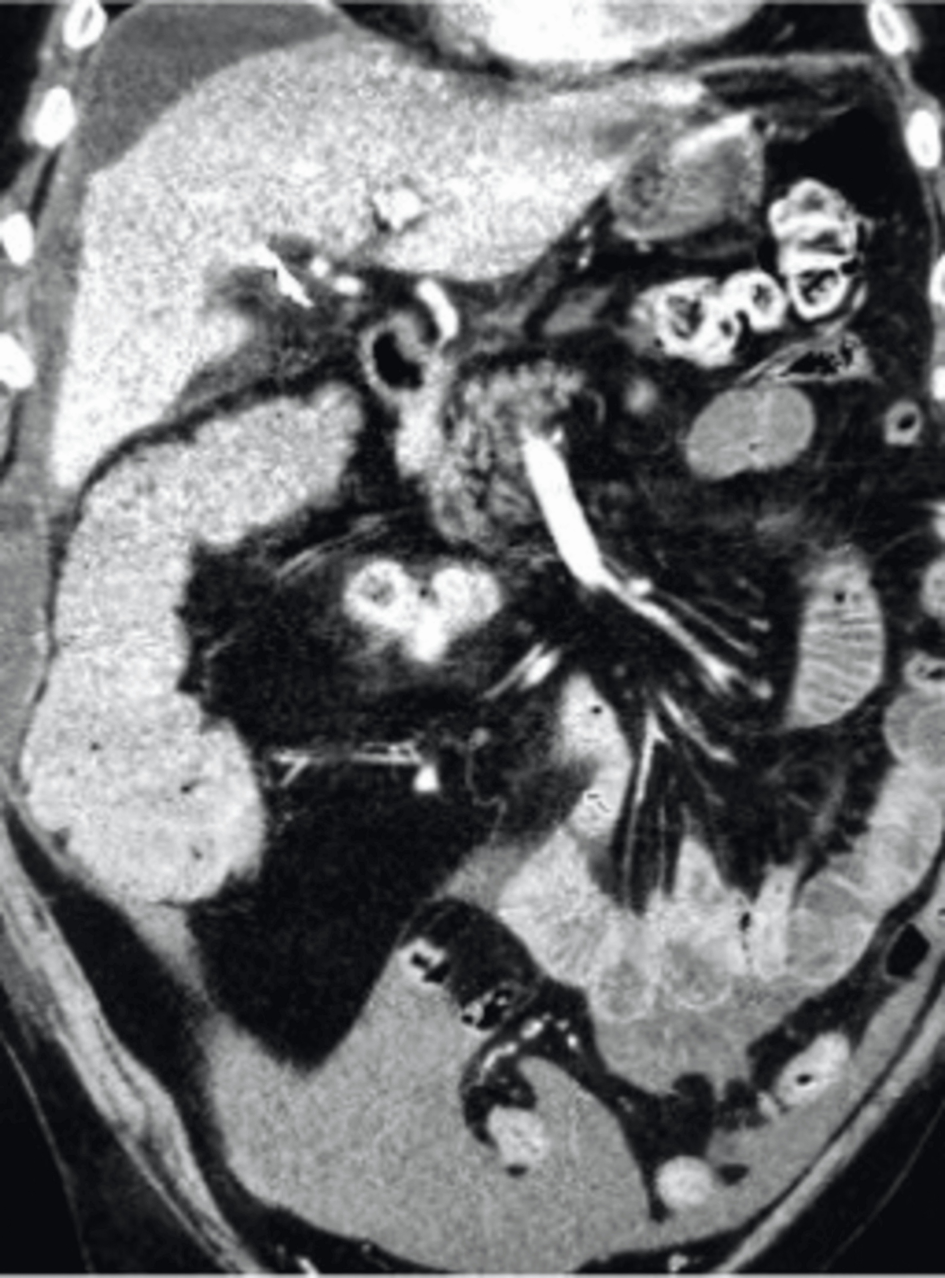
Fluid surrounding the liver and confirmation of surgical clip present at the site of cholecystectomy on CT imaging

**Figure 2:**
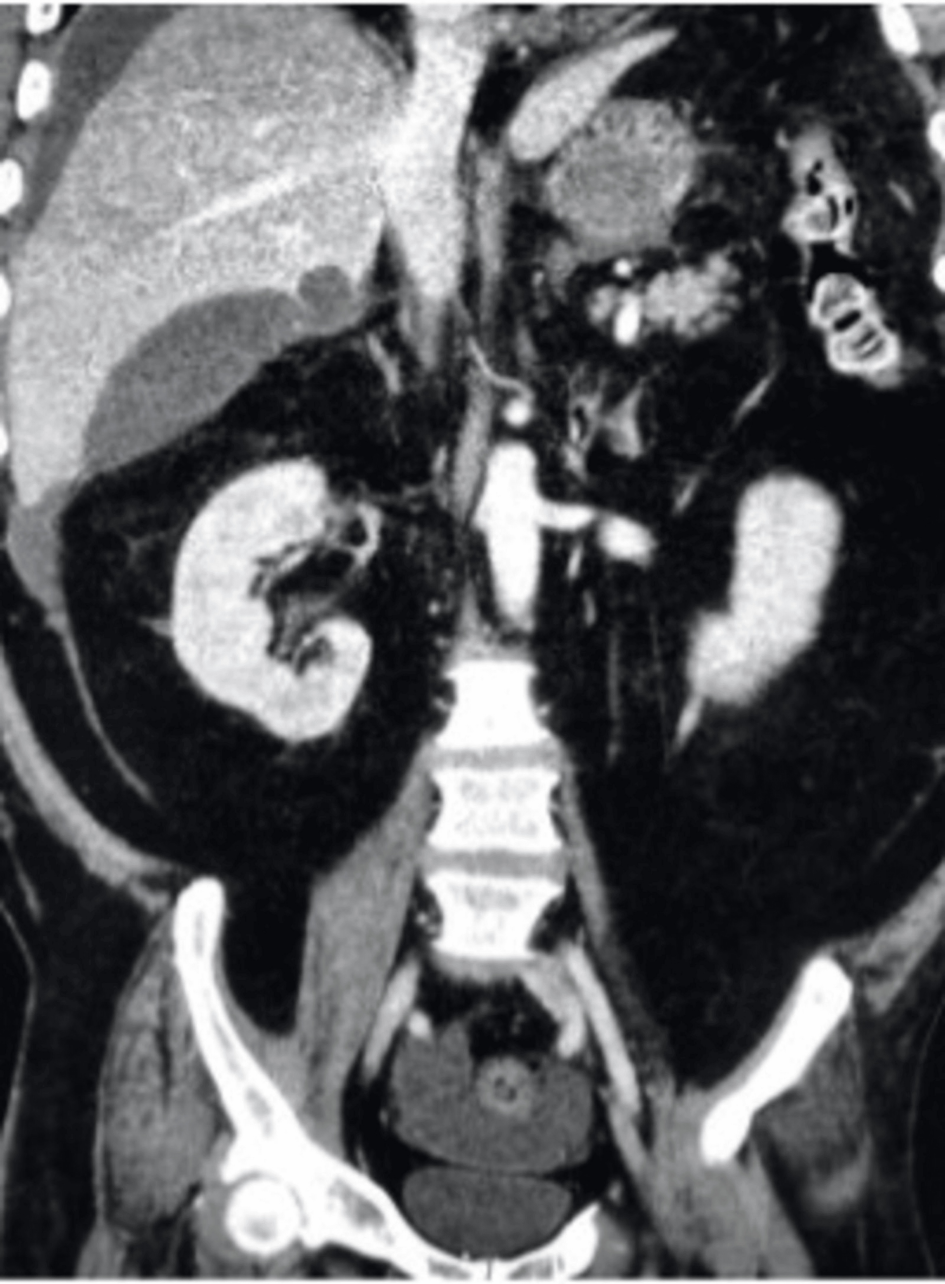
CT imaging showing a rounded or irregular low-density area (dark region) suggestive of fluid collection adjacent to the surgical site

We present a 54-year-old male, five days post-laparoscopic cholecystectomy, who presented to the emergency department with a one-day history of abdominal pain and fever. Physical examination revealed diffuse abdominal tenderness, with clean, dry surgical incisions showing mild surrounding erythema but no purulent discharge. Abdominal computed tomography (CT) imaging showed fluid accumulation around the liver and in the cystic duct, along with a fluid collection adjacent to the surgical site, suggesting a potential abscess (Figures 1, 2). In CT scans, an abscess generally appears as a fluid collection that may show up with a low density (darker area) compared to solid tissues, and it could have a rim of enhancement (brighter or whiter area) when contrast is used. The radiologist immediately communicated these findings to the emergency room physician.

**Figure 3:**
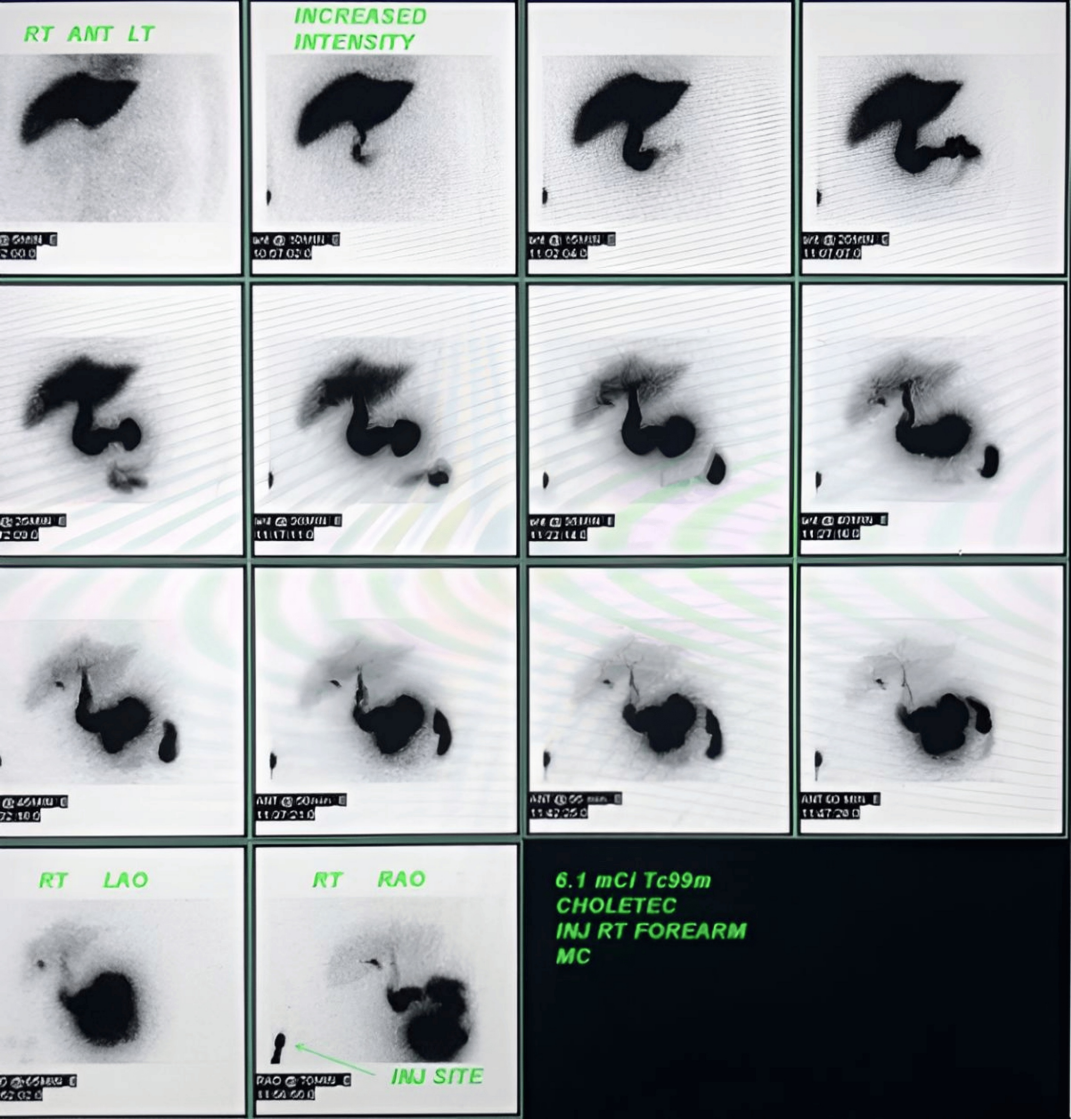
HIDA scan showing the gradual presence of abnormal accumulation of radiotracer outside of the biliary tree HIDA, hepatobiliary iminodiacetic acid

The patient was admitted based on the CT findings, and the operating surgeon was notified. A HIDA scan, as recommended by the radiologist, demonstrated normal liver, bile duct, and duodenum function. However, it also revealed a postoperative biloma with tamponading focal activity and no existing bile flow (Figure 3). A diagnosis of a bile leak from the duct of Luschka was made. The surgeon was informed, and a nonsurgical intervention was performed to close the duct.

## Discussion

This case report discusses a 54-year-old male who presented to the emergency department with abdominal pain and fever five days after undergoing laparoscopic cholecystectomy. Abdominal CT revealed biloma formation (Figures 1, 2), and a HIDA scan confirmed a bile leak from the ducts of Luschka (Figure 3). While the ducts of Luschka are present in a significant portion of the population, their clinical relevance often surfaces in middle-aged and older adults, especially women undergoing surgery for gallbladder disease [3-6]. Injury to these ducts during dissection in the gallbladder fossa can lead to bile leakage. Biloma formation post-cholecystectomy is uncommon, with our report being the fifth documented case in the literature [8-11]. Identifying a biloma post-cholecystectomy is crucial because it indicates a bile leak, which, if left untreated, can lead to severe complications such as infection, abscess formation, or sepsis, requiring timely intervention for optimal patient outcomes.

Complications involving the duct of Luschka have been reported following both cholecystectomy and liver transplantation [8,12]. A HIDA scan is a key diagnostic tool for bile duct injuries [13]. Bile leaks causing biloma formation can lead to symptoms such as diffuse abdominal pain and fever, as seen in this patient, or even an acute abdomen. Management of bile leaks generally involves nonsurgical interventions, such as endoscopic biliary stent placement. When bilomas are present, treatment typically includes percutaneous drainage, as was done for this patient. If conservative management fails, surgical intervention, including exploratory laparotomy, may be required. This procedure involves abdominal lavage, closure to the duct of Luschka, and intraoperative cholangiography to ensure the integrity of the biliary tree [7]. As the ducts of Luschka are anatomically variable and not consistently present in all patients, the case findings may not apply to all individuals undergoing cholecystectomy. This limits the broader application of the lessons learned from this case.

## Conclusions

Laparoscopic cholecystectomy has become the standard treatment for symptomatic cholelithiasis, replacing open surgery. While generally safe, complications can occur, particularly due to intraoperative injury to the ducts of Luschka, especially when unrecognized anatomic variations are present. This case reports a rare complication of biloma formation resulting from a bile leak following laparoscopic cholecystectomy in a patient with an undiagnosed ductal variation. The bile leak was confirmed using a HIDA scan and abdominal CT, and nonsurgical intervention was successfully employed to manage the leaking ducts of Luschka. This case, likely the fifth documented instance of biloma post-laparoscopic cholecystectomy, underscores the importance for surgeons to remain vigilant regarding anatomic variations in the ducts of Luschka, which can lead to postoperative complications such as bile leaks and biloma formation.

## References

[1] Spanos CP, Syrakos T (2006). Bile leaks from the duct of Luschka (subvesical duct): a review. Langenbecks Arch Surg.

[2] Keplinger KM, Bloomston M (2014). Anatomy and embryology of the biliary tract. Surg Clin North Am.

[3] Luschka H (1863). Die Anatomie des Menschen in Rücksicht auf die Bedürfnisse der praktischen Heilkunde bearbeitet. https://www.digitale-sammlungen.de/en/details/bsb10368862.

[4] Schnelldorfer T, Sarr MG, Adams DB (2012). What is the duct of Luschka? A systematic review. J Gastrointest Surg.

[5] Rosai J (2011). Rosai and Ackerman’s Surgical Pathology (10th ed.). https://shop.elsevier.com/books/rosai-and-ackermans-surgical-pathology-10e/rosai/978-81-312-2984-2.

[6] Handra-Luca A, Ben Romdhane HM, Hong SM (2020). Luschka ducts of the gallbladder in adults: case series report and review of the medical literature. Int J Surg Pathol.

[7] Ramia JM, Muffak K, Mansilla A, Villar J, Garrote D, Ferron JA (2005). Postlaparoscopic holecystectomy bile leak secondary to an accessory duct of Luschka. JSLS.

[8] Paramythiotis D, Moysidis M, Rafailidis V, Bangeas P, Karakatsanis A, Kalogera A, Michalopoulos A (2019). Ducts of Luschka as a rare cause of postoperative biloma. MRCP findings. Radiol Case Rep.

[9] Ishikawa M, Mizuguchi T, Suga M (2005). Progression and regression of biloma after laparoscopic cholecystectomy evaluated by computed tomography. Int Surg.

[10] AlNaqrani FA (2019). Biloma at the lesser sac post laparoscopic cholecystectomy. Int J Gen Med.

[11] Bátorfi J, Baranyay F, Simon E, Beznicza H, Kolonics G (2004). [aparoscopic treatment of bile leakage from the Luschka duct after laparoscopic cholecystectomy. Orv Hetil.

[12] Vilar Tabanera A, Puerta Vicente A, López Buenadicha A, Peromingo R, Lopez Hervás P, Nuño Vasquez-Garza J (2020). Luschka duct leak: An unexpected cause of choleperitoneum after liver transplant. Exp Clin Transplant.

[13] Kousik V, Bhattacharya A, Yadav TD, Mittal BR (2020). Hepatobiliary scintigraphy-role in preliminary diagnosis and management of biliary tract injuries. Clin Nucl Med.

